# Editorial: Obesogens in the XXI century: Emerging health challenges

**DOI:** 10.3389/fendo.2022.999908

**Published:** 2022-08-08

**Authors:** Ana C. A. Sousa, Govindan Malarvannan, Tomohiko Isobe, Luís Rato

**Affiliations:** ^1^ Department of Biology, School of Science and Technology, University of Évora, Évora, Portugal; ^2^ Comprehensive Health Research Centre (CHRC), University of Évora, Évora, Portugal; ^3^ Toxicological Centre, Department of Pharmaceutical Sciences, University of Antwerp, Wilrijk, Belgium; ^4^ Japan Environment and Children’s Study Programme Office, National Institute for Environmental Studies, Ibaraki, Japan; ^5^ CICS – UBI – Health Sciences Research Centre, University of Beira Interior, Covilhã, Portugal; ^6^ Health School of the Polytechnic Institute of Guarda, Guarda, Portugal

**Keywords:** endocrine-disrupting chemicals (EDC), obesogens, non-communicable diseases (NCD), obesity, overweight

The global prevalence of overweight and obesity has risen dramatically in recent decades. Obesity is a major public health problem, recognized by the World Health Organization as one of the most important public health challenges of the 21^st^ century (1). Therefore, preventing obesity is a public health priority for adults, children, and adolescents. This is especially important because overweight and obese children are likely to remain obese into adulthood and are more likely to develop non-communicable diseases such as diabetes and cardiovascular disease at a younger age. Several factors are thought to be involved in the obesity pandemic, but in recent years the focus has been on exposure to specific environmental pollutants, the obesogens. The obesogen hypothesis was postulated in 2006 by Grün and Blumberg (2). The authors found that tributyltin could induce adipogenesis *in vitro* and *in vivo*. This “simple” finding was a huge breakthrough in Endocrinology and Metabolism. In these almost two decades, a significant body of evidence was gathered, and currently, obesogens have been considered key actors in the obesity epidemic.

Obesogens can disrupt hormonal pathways regulating metabolism and weight gain (2). Over our lifetime, chronic exposure to obesogens is likely the leading cause of obesity and metabolic disorders, costing hundreds of billions of euros each year (3). Therefore, assessing exposure to these chemicals and understanding their impact on human health is pivotal to developing strategies to reduce vulnerability and improve health. Furthermore, molecular understanding of the toxicity induced by obesogens is pivotal to develop strategies to provide better diagnostic platforms, treatment, and management of obesogenic side effects.

In this Research Topic, we aimed to cover all aspects of the obesogen field, from exposome (4) to the effects on model organisms and humans (5, 6), including the study of the long-term impacts of obesogen exposure.

The data gathered over the last years has demonstrated that the effects of obesogen exposure can be more pronounced than what was previously thought. Their effects can be passed from one generation to another, *i.e.*, transgenerationally. Blumberg’s team has extensively addressed this topic and in the review by Mohajer et al. they discuss the mechanisms underlying transgenerational inheritance. They propose that changes in our chromatin organization and structure may be a plausible explanation for how some disease predispositions can be passed throughout multiple generations, including those that were not exposed.

Tributyltin is considered “the obesogen model” (4), yet many other EDCs have also been ascribed as obesogens. These chemicals are ubiquitous, being found in several products of our daily life and along the food chain. The current epidemiological evidence of the obesogenic effects of different EDCs is reviewed by Mohanto et al. The authors compiled the available information on the associations between EDCs levels in the blood and/or urine samples and overweight/obesity indices across all life stages. This review is a relevant contribution to the obesogen field highlighting the obesogenic effects of EDCs among the general population. The gathered evidence reveals consistent obesogenic roles of BPA, DDE, and PFOA, but inconsistent roles of phthalate metabolites and other POPs, stressing the need for further studies. Besides the traditional EDCs addressed in this review, there is increasing evidence that other environmental contaminants might display obesogenic potential. Kannan and Vimalkumar present an exciting review about permanent human exposure to microplastics (MPs) and how MPs act as obesogens. This study provides up-to-date information on the sources and pathways of human exposure to MPs and their toxic effects, especially those related to obesogenic mechanisms of MPs toxicity.

Although essential determinants to the obesity epidemic, obesogens are not the only culprits, with lifestyle and socioeconomic and nutritional status also playing a major role. Our eating habits dictate if we are or are not at high risk of developing obesity. Circulating levels of adipocytes-related hormones are frequently altered in obesity (5, 6). For instance, leptin and adiponectin play a role in weight regulation and treatment outcomes. Leptin is responsible for regulating appetite and satiety, food intake, energy homeostasis, and body fat since it acts in the central nervous system, in the hypothalamus. Obese people present increased levels of leptin, whereas adiponectin production is reduced. In this line, Vermeiren et al elegantly determined the predictive value of baseline patient characteristics (age, sex, and adiposity) extended with cardiometabolic comorbidities, leptin, and adiponectin in early (during treatment) and late (during aftercare) dropouts and short- and long-term treatment outcomes in children with obesity treated in an inpatient pediatric obesity treatment program. The authors reported for the first time that the pre-treatment levels of leptin and adiponectin predict post-treatment BMI SDS regain, which opens the door to further research in this exciting field. Overall, this study highlights that the patients needing treatment the most are at higher risk for dropouts and weight regain, reinforcing the need to implement intervention programs to help patients after discharge to reduce dropouts.

Although the prevalence of overweight and obesity worldwide is high, it may differ between regions, with poor areas showing the highest incidence and prevalence rates. Apart from genetic and hormonal dysregulation, the socio-economical status (SES) is also an important player in the aetiology of the disease. A low SES often dictates the health profile of the person. In this sense, Massicard et al. estimated the incidence of obesity and overweight in different populations of French Guiana, highlighting the relevance of socio-economic and nutritional status in this process.

The impacts of obesity are diverse and include the development of several comorbidities such as type 2 diabetes mellitus (T2DM), hypertension and cardiovascular diseases. Gao et al. evaluated the association between obesity and microvascular diseases in patients with T2DM. In this work, the authors compared the magnitude of several parameters such as fat mass index (FMI), body mass index (BMI) and waist circumference (WC) with the risk of microvascular diseases to find the best indicator of obesity- associated with risk of microvascular diseases among patients with T2DM. After the evaluation of the chronic kidney disease (CKD) progression, retinopathy, and neuropathy as primary microvascular outcomes, the authors concluded that obesity is associated with CKD progression and neuropathy in T2DM participants. They further showed that in T2DM patients, FMI and WC are more valuable in identifying the obesity-related risk of neuropathy.

Several aspects of the association between weight gain and vascular problems remain to be clarified. Association between obesity and vascular problems affects numerous individuals in the population, mainly women, during sensitive periods, such as pregnancy. In this context, the original research performed by Wu et al. has demonstrated that the associations between gestational weight gain and the category of venous thromboembolism (VTE), pulmonary embolism (PE) or deep venous thrombosis (DVT) with or without PE vary with the different periods of pregnancy. This work brings new evidence on how it is important to manage weight gain and venous thromboembolism during gestational periods.

By addressing obesity from different perspectives, *i.e.*, from the molecular level to the population level, from the possible causes to the consequences, we hope that this article collection contributes to improving our knowledge of this public health priority and may open new perspectives on this complex topic.

## Author contributions

AS, GM, TI, and LR participated in the design of the manuscript, analyzed the bibliographic data, collected them, and drafted the manuscript. All authors critically revised the manuscript. All authors contributed to the article and approved the submitted version.

## Funding

This work was developed within the scope of the CHRC project UIDP/04923/2020 and CICS-UBI projects UIDB/00709/2020 and UIDP/00709/2020, financed by national funds through the Portuguese Foundation for Science and Technology/MCTES.

## Conflict of interest

The authors declare that the research was conducted in the absence of any commercial or financial relationships that could be construed as a potential conflict of interest.

## Publisher’s note

All claims expressed in this article are solely those of the authors and do not necessarily represent those of their affiliated organizations, or those of the publisher, the editors and the reviewers. Any product that may be evaluated in this article, or claim that may be made by its manufacturer, is not guaranteed or endorsed by the publisher.
